# Hydrogels as Suitable miRNA Delivery Systems: A Review

**DOI:** 10.3390/polym17070915

**Published:** 2025-03-28

**Authors:** Haseena Makada, Moganavelli Singh

**Affiliations:** Nano-Gene and Drug Delivery Laboratory, Discipline of Biochemistry, University of KwaZulu-Natal, Private Bag X54001, Durban 4000, South Africa; 217003524@stuukznac.onmicrosoft.com

**Keywords:** miRNA, hydrogels, polymers, delivery, drug development, carrier systems

## Abstract

The use of miRNA in therapeutics has, since its discovery in 1993, attracted tremendous attention, and research in this area has progressed rapidly. Since the advent of RNA interference (RNAi), much about the nucleic acid siRNA has been elucidated. At the same time, no miRNA-based drugs have passed phase II clinical trials. A significant obstacle to miRNA-based drug development is the ease of degradation and relatively short half-life in vivo of miRNA. Hydrogels are networks of cross-linked polymer chains with the ability to ‘swell’. They have shown remarkable capabilities that improve the properties of other researched carriers. In combination with miRNA modification strategies and inorganic carriers, hydrogel systems show promise for sustained miRNA delivery and the development of novel miRNA-based drugs. Although hydrogel systems have been reported recently, the focus has been predominantly on their wound-healing properties, with a dearth of information on their nucleic acid carrier abilities. This paper focuses more on the latest advancements in developing hydrogels as a carrier system, emphasizing the delivery of miRNA. This review will cover the methods of hydrogel fabrication, efforts for sustained miRNA release, biomedical applications, and future prospects.

## 1. Introduction

The short non-coding RNA molecules called microRNA (miRNA) were first discovered in a roundworm species in 1993. Currently, the number of mature miRNAs estimated to be present in human cells is 2588, and they are reported to control over 60% of the gene expression [1]. miRNAs function in gene silencing through direct interaction with messenger RNA (mRNA), causing destabilization or degradation of mRNA and inhibition of protein synthesis. miRNAs associate with a multiprotein complex termed the RNA-induced silencing complex (RISC) to typically bind the 3′ untranslated region (3′UTR) and, less commonly, the 5′ untranslated region (5′UTR) or coding region of target mRNA through sequence complementarity. Endogenous miRNAs are responsible for various biological processes such as cell proliferation and differentiation, angiogenesis, migration, and cell death [2]. miRNAs also demonstrate clear expression patterns in multiple tissues during different developmental stages of human diseases. For example, miR-21 is a diagnostic marker in several types of cancer, such as breast and lung cancer, while miR-222 is a diagnostic marker in ovarian and non-small-cell lung cancer (NSCLC). miR-21 is a prognostic marker in lung, gastric, and liver cancer [3]. miRNAs are strong regulators of gene expression and can interact with multiple target genes. They are explored as therapeutic agents in various pathological diseases, including cancer, as they can restore disrupted cellular functions.

A significant obstacle in miRNA therapeutics is what has been described as ‘too many targets for miRNA effect’ (TMTME) [4]. This is the typical miRNA binding to various suitable sequences because of imperfect complementation with target sequences. Hence, a single miRNA strand can have hundreds of unapproved targets, which is unsuitable due to off-target effects. The therapeutic Miraversen developed for treating hepatitis C virus (HCV) infection demonstrated significant efficacy. This miRNA-based drug underwent several phase II clinical trials only to be terminated due to severe side effects [1]. Interestingly, Zhang and coworkers reported that only 10 miRNA drugs have been undergoing clinical trials, with none reaching phase III [4]. This is comparable to siRNA-based drugs, where patisiran and givosiran have already received FDA approval. This underlines the importance of creating targeted and sustainable miRNA delivery systems.

The delivery of naked miRNA in vitro and in vivo is generally inefficient due to nuclease susceptibility, lysosomal degradation, low cell membrane permeability, and rapid renal clearance. These issues demand high doses of RNA, which increases the risk of non-specific interactions. These challenges have necessitated the introduction of delivery vehicles, which have also been extensively researched over the years. Viral vectors, lipids, polymers, extracellular vesicles, and nanoparticles (NPs) have all been used in miRNA delivery [5]. While these vehicles overcome many of the challenges of naked miRNA, they still cannot guarantee prolonged action in vivo [6]. Chemical modifications such as the cholesterol modification of miRNA can overcome cell repulsion through passive internalization in vitro and in vivo [7]. However, rapid renal clearance and short half-life are still issues faced by chemically modified miRNA [6].

**Figure 1:**
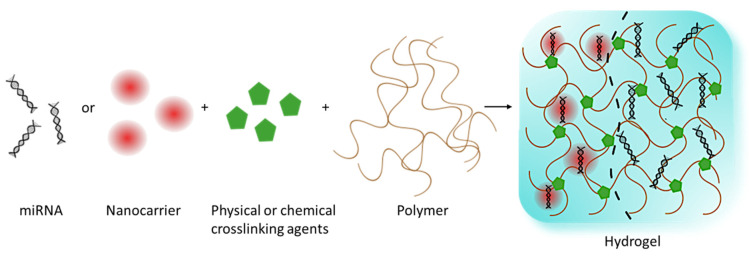
Schematic illustration of basic hydrogel formation (drawn using Inkscape).

Hydrogels are networks of cross-linked polymer chains that retain water within their structure but do not dissolve in water, allowing them to ‘swell’. The final macro-molecular structure is that of a three-dimensional gel, which can swell several times its dry weight [8]. The materials from which hydrogels are made can be either natural or synthetic polymers, each having its advantages [9]. Natural polymers such as alginate [10], chitosan [11,12], and cellulose [13] have good renewability, biodegradability, and biocompatibility. Synthetic polymers such as polyacrylamide [14], polyethyleneimine [6], polyethylene glycol [15], and poly(vinyl alcohol) [16,17] became increasingly popular due to increased strength and stability, ease of functionality, and higher water absorption [18]. They may, however, have undesirable cytotoxicity [19]. Peptide-based hydrogels are an emerging class of biomaterials formed through a multifaceted self-assembly approach dependent on the amino acid sequence from which they are formed [20]. Hydrogels generally suffer from low mechanical strength, a property required in tissue engineering applications, which subsequently gave rise to nanocomposite hydrogels. This will be discussed later on in this review. The type of material selected should align with the individual research objectives and the intended application. For example, hydrogels with increased mechanical strength would be employed in wound healing assays, and biodegradable materials would be selected to treat diseases. The cross-linking step in hydrogel fabrication can be used to classify hydrogels, with physical and chemical cross-linking being the two main processes (Figure 1). Chemical cross-linking involves the formation of covalent bonds, and physical cross-linking occurs via hydrogen bonding and hydrophobic or ionic interactions [21].

In cancer treatment, hydrogel-mediated delivery of miRNA showed increased tumor reduction compared to doxorubicin and paclitaxel. Furthermore, these traditional chemotherapeutic agents are often associated with tumor recurrence after treatment, necessitating multiple administrations [22]. In tissue engineering, hydrogels provide a favorable microenvironment to support regeneration after initial delivery. Their ability to take on the shape of defects also makes them a robust solution [23]. In cardiac diseases such as myocardial infarction, hydrogel-based intramyocardial delivery of miRNA exhibits improved cardiac efficacy, increasing cardiomyocyte proliferation [15]. This review will focus on the recent advances made in hydrogel systems for miRNA delivery and the applications for which they have been used.

## 2. Hydrogel Formulation

**Figure 2:**
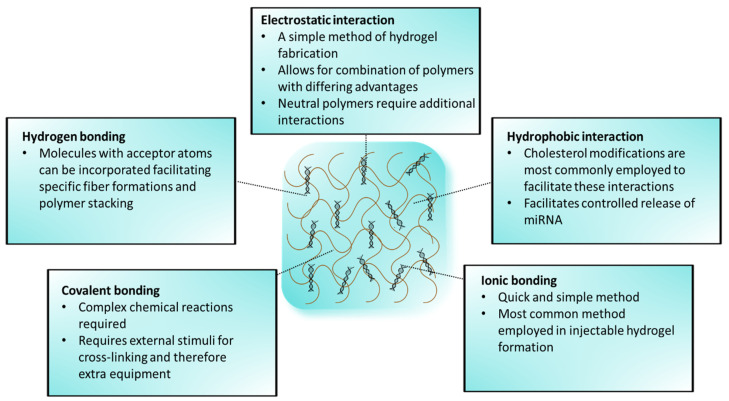
Approaches for hydrogel fabrication and miRNA loading.

There are several approaches in which hydrogels can be synthesized (Figure 2).

### 2.1. Electrostatic Interactions

Electrostatics is an important theme when considering materials in polymer selection and presents as another way of classifying polymers. The negative charge on miRNA generally calls for a polymer with a cationic or neutral charge for encapsulation and effective release from the hydrogel. The anionic polymer alginate with the cationic polyethyleneimine (PEI) was reported to form a stable, non-toxic, and biocompatible hydrogel that exhibited targeted miRNA delivery and slow release [24]. Cationic polymers such as chitosan and the polyamidoamine (PAMAM) G5 dendrimer easily facilitate electrostatic interactions for attaching negatively charged miRNA [22,25]. However, many hydrogel systems are formed from more than one polymer, conferring varying advantageous properties for successful gene delivery. Polymers such as gelatin, derived from collagen, are commonly modified with methacryloyl groups, forming methacrylate gelatin (GelMA) that can be either cationic or anionic [26,27].

### 2.2. Covalent Bonding

Two types of chemical cross-linking are commonly seen in hydrogel formation: covalent and non-covalent. Covalent cross-linking requires external stimuli such as light, pH, and temperature change for initiation, which also controls miRNA release from the hydrogel [27]. The photo-click reaction is a covalent cross-linking approach in which a photoinitiator is added to the reaction. This facile method was used to couple the polymers gelatin-norbornene and PEG-dithiol in a thiol-ene reaction exhibiting gradual miRNA diffusion and enhanced osteogenesis [23]. The disadvantage of this method in biomedical applications, especially in drug delivery, is that drugs can be lost during the network formation [28]. Furthermore, external equipment, such as a UV-light source, is required.

### 2.3. Non-Covalent Interactions

Non-covalent cross-linking, such as hydrophobic interactions, Schiff interactions, and ionic cross-linking, follows a much simpler path of gel formation since an external trigger is not required, and formation is generally quick [29,30]. Ionic cross-linking employs cations to associate chains of polymers to be ‘glued’ together at junction zones. Divalent calcium cations (Ca^2+^) are commonly used as the cross-linking agent for alginate hydrogels [31,32,33]. Injectable hydrogels are commonly formed through non-covalent interactions and exhibit shear thinning in which the hydrogel viscosity decreases with increased shear stress [34]. For medicinal applications, injectable hydrogels are a research hotspot, as they can overcome many obstacles that other miRNA delivery systems face. Local miRNA delivery achieved by injectable hydrogels allows for enhanced cellular uptake and reduces toxicity associated with non-specific uptake in healthy tissues [35]. Clinically, pre-crosslinked hydrogels would require surgical implantation, which is invasive. However, shear thinning allows hydrogels to be injected through a syringe directly into the defect site, which is much less invasive. Under shear thinning, physical cross-links are reversible [7]. The naturally occurring glycosaminoglycan hyaluronic acid (HA) has been used to design injectable hydrogels. Xu and coworkers developed an injectable hydrogel based on a simple guest–host assembly mechanism [36]. HA modified with β-cyclodextrin, and HA modified with adamantane were mixed to form the final product, achieving localized miRNA delivery. Spontaneous formation of an amine-modified HA-based hydrogel was described where an NHS-terminated 8-arm-PEG cross-linker was mixed into solution [35]. Although less common, injectable hydrogels can also be formed through covalent interactions. PEG hydrogels with encapsulated miRNA polyplex NPs were formed by mixing equimolar aqueous solutions of 4-arm-PEG-SH and 4-arm-PEG-MAL, each containing the miRNA polyplex [6].

UPy-PEG hydrogels, which are PEG strands functionalized with ureido-pyrimidine moieties through hydrogen bonding, show promise in miRNA delivery [15]. The urea groups facilitate lateral stacking, allowing for cross-linking and fiber formation. A positively charged hydrogel is obtained for miRNA delivery by adding an oligo-ethylene glycol (OEG) functionalized with an amine group on the UPy moiety. This gel type is useful in in vivo studies due to its pH responsiveness and reversible nature. In cardiac applications, this hydrogel induces transient inflammatory responses and exhibits low retention at the injection site.

A decellularized extracellular matrix (ECM) is a naturally derived biomaterial that can be delivered via catheter or injection and is minimally invasive. Unlike other hydrogels, this platform uses only natural materials without chemical modifications or additional cross-linkers. Porcine-derived ECM hydrogels decellularized with sodium dodecyl sulfate (SDS) detergent exhibited sustained miRNA release and enhanced cellular uptake [37]. Silk fibroin is another biomaterial extracted from silkworm cocoons that mechanically matches the properties of the ECM. It undergoes an easy gelling process at 37 °C without a cross-linker [38]. In another study, self-assembling peptides (SAPs) integrated with miRNA that underwent gelation were found to be promising material that mimicked the ECM and provided sustained cargo delivery [39]. Exosomes have also become increasingly popular components of hydrogel systems [26,38,40]. Exosomes are vesicles with sizes in the nanometer range that can target the ECM and promote balance as well as chondrocyte regeneration [40]. Unfortunately, due to their short half-life, exosomes must be incorporated into hydrogel systems, which function as supporting matrices that promote sustained release [38].

### 2.4. Nanocomposite Hydrogels

NPs have been included in hydrogel systems to increase biostability and increase miRNA protection [28]. They also play a part in the release kinetics of miRNAs within hydrogels. In nanocomposite hydrogel systems, miRNAs are functionalized onto NPs through electrostatic interactions before being loaded onto the hydrogel [41]. However, nanocomposite hydrogels have not yet exceeded Lipofectamine transfection efficiency, supposedly due to its complete optimization. PNP (polymer–NP) interactions are a non-covalent and reversible form of injectable hydrogel synthesis. This method was reported where a hydroxypropylmethylcellulose (HPMC-C_12_) polymer and poly (ethylene glycol)-block-poly (lactic acid) (PEG-*b*-PLA) NPs were mixed to form a hydrogel that was further loaded with miRNA nanocomplexes. The NPs were formed via nanoprecipitation, and the resulting gel was formed by polymer bridging over multiple NPs [28]. Saleh and coworkers showed that miRNA release rates increased with increased NP concentration, implying that miRNA release could be controlled [42].

RNA nanohydrogels are another type of hydrogel formed without additional polymers. RNA nanohydrogels have exhibited efficient cellular uptake, enhanced biocompatibility, and nuclease resistance [19]. These gels are formed from the self-assembly of polymerized RNAs into microsponges based on rolling circle transcription (RCT). Among other tunable properties, these gels can contain multiple miRNA types, all targeting different genes concurrently. This creates a multifunctional synergistic treatment option [19,43]. Incorporating carbon dots in hydrogels is particularly interesting, as they serve as fluorophores, imparting good fluorescent features to the hydrogel and as the cross-linker [44]. Recently, RNA nanohydrogels have been combined with chemotherapeutic agents and NPs in a novel strategy to overcome therapeutic limitations. Wang and coworkers developed a synergistic combination therapy involving photodynamic therapy, chemotherapy, and gene delivery via hydrogel to treat triple-negative breast cancer (TNBC) in MDA-MB-231 cells [41]. This type of treatment overcomes the poor cellular uptake, poor bioavailability, and resistance associated with conventional therapy for TNBCs. RNA hydrogels can also be formed through an emulsification process involving grafting nucleic acids onto polymer hybrids in the presence of co-crosslinkers [45]. This method allows for the incorporation of additional elements, such as chemotherapeutic drugs for a combined therapeutic result and fluorophores for bioimaging applications.

## 3. Sustained Release/Stimuli-Responsive Gels

A favorable property in hydrogels is the ability to precisely control the release of the miRNA strand since a conservative release would not achieve the desired effect, and excessive release could cause damage to healthy tissue [6]. Release of miRNA from hydrogels generally follows two phases: an initial rapid release due to hydrogel swelling followed by a slower, sustained release. The second phase of miRNA release and diffusion is attributed to gradual hydrogel degradation [35]. The dissolution of hydrogel scaffolds is closely related to miRNA release profiles and is an important consideration when designing hydrogel networks [33,36,40].

### 3.1. Active Release

The incorporation of stimuli-responsive linkages, such as ultraviolet (UV) light-cleavable linker lithium phenyl-2,4,6-trimethylbenzoylphosphinate (LAP) [23], into the hydrogel system is one mechanism for controlling miRNA release [43]. When exposed to UV irradiation, the hydrogel can shift from a gel into a solution, releasing miRNA at an enhanced rate. If the miRNA is modified directly onto the backbone of the hydrogelator as opposed to encapsulation within the matrix, it prevents the unwanted or premature release of miRNA. Notably, the addition of photosensitive groups increases the thickness of the gel, which may affect its properties [46]. Another limitation of this strategy is that UV light is impenetrable, implying that the system must be controlled in vitro before being released in vivo.

Redox-responsive hydrogels are another class of hydrogels employed for precise miRNA release. Large amounts of endogenous glutathione (GSH) are produced as an intermediate metabolite in the affected microenvironments [47], which is exploited as a mechanism of miRNA release. Excess GSH at tumor sites can break some of the bonds responsible for hydrogel integrity, resulting in hydrogel degradation and miRNA release [45]. Lei and coworkers fabricated mesoporous silica NPs (MSNs) modified with disulfide bonds carrying miRNA that were degraded by GSH upon endocytosis, subsequently allowing for the release of the miRNA [48]. This type of setup allows for prolonged miRNA transfection. A similar strategy was employed in treating myocardial infarction, where intact miRNA was released, indicating strong feasibility [47]. Thermoresponsive hydrogels are engineered to change viscosity with a temperature change [48]. Temperature-sensitive di (ethylene glycol) methyl ether methacrylate (DEGMA) has been grafted onto polymers to achieve thermoresponsive hydrogels. Hydrogels can be designed to be in a solid state at low temperatures and a gel state at 37 °C so that the sol–gel transition occurs upon skin contact in the case of topical application [49]. Other hydrogels have been designed to be liquid at room temperature and a gel upon contact with the affected area. The liquid state allows easy administration, and the gel state increases retention time. Poly (lactic-co-glycolic acid) (PLGA) is another polymer employed to confer thermosensitivity due to its glass transition temperature being higher than the body’s temperature [25]. The copolymer poly (N-isopropyl acrylamide) (PNIPAM) allows for a reversible change to occur where the sol–gel transition can be recurrently induced at different temperatures [48].

Environment-responsive hydrogels exploit the specific characteristics of the disease-affected microenvironments. For example, local acidification is seen in inflamed microenvironments caused by degenerative disorders [50]. Increased acidity or alkalinity requires a unique subset of pH-responsive hydrogels [30]. Glycidyl methacrylate (GMC) is a pH-responsive polymer allowing targeted miRNA release [40]. Matrix metalloproteinases (MMPs)-responsive hydrogels are another subset of hydrogels responding to an increased MMP presence in various tissues to release miRNA. The MMP-sensitive peptides incorporated into the PEG hydrogel system were shown to produce a timed sequential release of miRNA cargo [27,51].

### 3.2. Passive Release

The cholesterol modification of miRNA is one strategy that can be used to control release within hydrogels. Highly hydrophobic cholesterol-modified miRNA is thought to have an affinity for the water-shielded hydrophobic interior of hydrogels, preventing a fast release of miRNA. Release profiles involving cholesterol-modified miRNA show initial repressed burst release followed by a steady linear rate to complete release [52]. Agomirs (cholesterol-, methylation-, and phosphorothioate-modified miRNA mimics) are commonly employed in hydrogel systems for increased cellular affinity and improved transfection efficiency. These chemical modifications also enhance stability, thereby reducing the need for extra protection measures that can be costly. Some risks of these modifications include leakage and reduced efficacy due to easy diffusion in deep tissues [29]. Generally, the non-modified miRNA exhibits much faster release [7]. However, cholesterol modifications are limited by the viscosity increase seen upon adding miR-chol, which hinders injectability.

The concentration of the hydrogelator is also thought to affect the nucleic acid release profile. This provides an additional mechanism for controlling release. In UPy-PEG hydrogels, increased hydrogelator concentration showed reduced erosion and lower initial burst releases [52]. One hypothesis is that lower cross-linking density allows for increased mobility and faster diffusion of miRNA out of the hydrogel [23]. Similar results were seen in gelatin-alginate hydrogels where increased cross-linking decreased the rate and amount of miRNA released [32]. The degradation study also showed that increased cross-linking degree decreased the degradation rate, directly influencing the miRNA release rate [53]. It is important to note that multiple strategies to facilitate miRNA release in a single hydrogel system can enhance its synergism and efficiency.

Another strategy used in release mechanisms is the addition of a cationic moiety within the hydrogel to facilitate electrostatic interactions with miRNA, thereby delaying release. Research shows a direct inverse correlation between the number of cationic charges and the additive and cumulative miRNA release. With highly positive hydrogels, strong electrostatic affinity can prevent the release of miRNA from the hydrogel [44,52,54]. In both these strategies, release can be fine-tuned.

**Figure 3:**
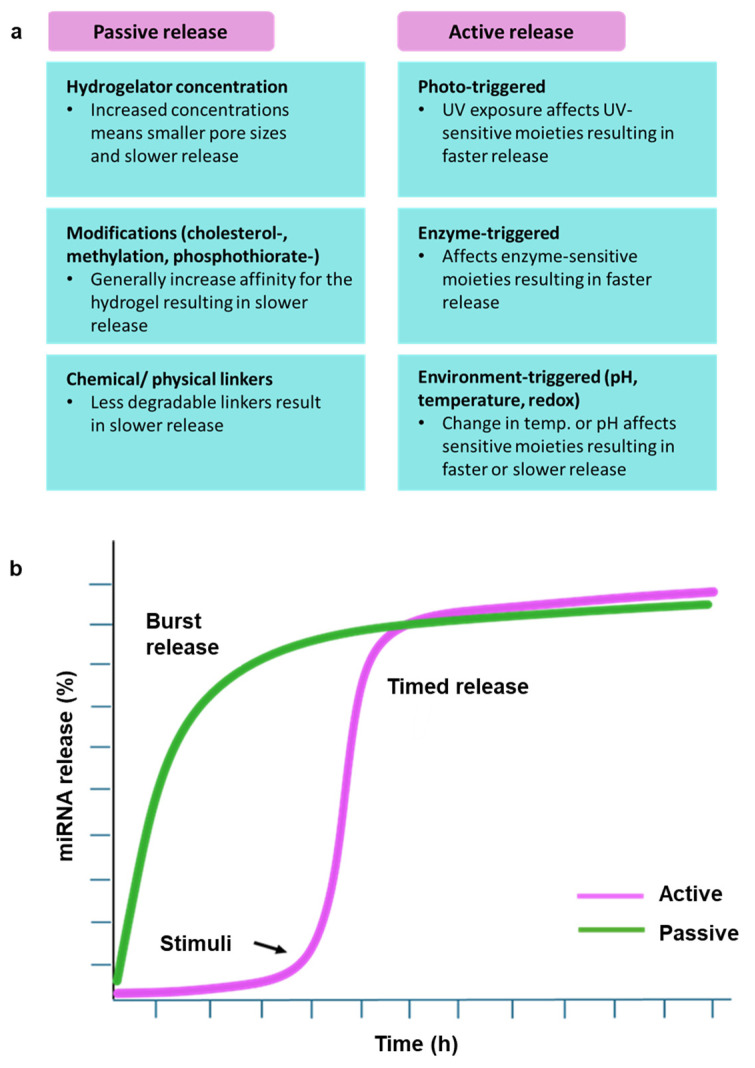
(**a**) Active and passive release of miRNA from hydrogel systems. (**b**) Release profiles of naked miRNA within hydrogel systems.

The two major release systems of hydrogels, their subsets, and their release profiles are summarized in Figure 3.

## 4. Hydrogel Optimizations

Effective hydrogel design depends on the choice of polymer, cross-linking degree, biocompatibility, and biodegradability. These factors significantly influence miRNA stability and release. This section looks at how different materials behave in vivo and discusses the commercialization of hydrogel systems.

### 4.1. Stability, Release, and Cytotoxicity

Chitosan is a biocompatible and biodegradable polymer suitable for hydrogel formation. However, chitosan hydrogels are commonly enhanced with nanoparticles to improve stability. In treating allergic rhinitis, PEG-PLA NPs were functionalized onto the chitosan hydrogel to reduce the risk of instability due to the enrichment of enzymes in the nasal cavity [25]. In another study, a chitosan hydrogel was loaded with PEG-PEI-graphene oxide NPs carrying miRNA [53]. Notably, the PEI presented with cytotoxicity at a high concentration of 50 μg/mL. While the hydrogel system exhibited in vivo bone generation, the chitosan degraded faster than the bone regeneration rate. This underlines the downside to the good biodegradability of natural polymers. PEI cytotoxicity was also seen in collagen hydrogels carrying PEI-functionalized ceria nanoclusters [55]. In this case, the ceria nanocluster could scavenge increased ROS species, thereby lowering cytotoxicity.

Cellulose is a polysaccharide whose derivatives are biodegradable and biocompatible [45]. PEG-PLA NPs were encapsulated within a HPMC-C_12_ hydrogel and further loaded with gold nanoparticles carrying miRNA to form PNP hydrogels [28]. Furthermore, the HA1 peptide was added to increase miRNA uptake through endosomal membrane destabilization. Previous studies have shown these PNP hydrogels to be highly viscoelastic with shear thinning due to hydrophobic cross-linking [56]. Hydrogels can be sprayed onto affected tissue in cardiac applications and flow to cover irregular surfaces [57].

Alginate-PEI hydrogels exhibit good cell viability in normal liver cell lines and HCC lines as well as good stability and sustained release [24]. This might indicate that alginate can mitigate the cytotoxic effects of PEI. Gelatin-alginate hydrogels exhibited good biocompatibility in mesenchymal stromal cells (MSCs) [32]. In vivo, degradation studies indicated that hydrogels with higher degrees of cross-linking facilitate cell adhesion and maintain their 3D structure with prolonged implantation. Conversely, hydrogels with lower degrees of cross-linking could not provide substantial mechanical support for tissue growth.

Hyaluronic acid (HA) hydrogels are useful in tissue regeneration applications since they can mimic the extracellular matrix (ECM) so closely [58]. Β-cyclodextrin-modified HA hydrogels showed good biocompatibility and localization to the affected area [36]. Matrix dissolution and physical diffusion were the mechanisms for release. Β-cyclodextrin is also reported to interact with cholesterol-modified miRNA to aid in slower release [7]. HA hydrogels also showed antibacterial properties, making them useful in wound healing applications [59].

Pluronics are copolymers comprising polypropylene glycol (PPG) and PEG in linear sequences [60]. They are injectable, thermoresponsive hydrogels that have been well-studied in tissue engineering applications. Although they are biocompatible, they are not biodegradable, necessitating the introduction of a biodegradable moiety [61]. Dai and coworkers reported a sodium alginate-pluronic F-127 hydrogel with complete miRNA release over 36 days and around 70% degradation at 30 days [31].

PEG is one of the most common polymers used in hydrogel synthesis. PEG hydrogels are hydrophilic and non-ionic, providing increased stability of encapsulated miRNAs through reduced interactions with serum proteins. PEG photodegradable hydrogels formed through ‘click’ chemistry showed no cytotoxicity or immune responses. The miRNA release was UV-triggered, and cholesterol modifications enhanced cellular entry [46]. PEG-PLGA-PNIPAM hydrogels showed temperature-dependent sustained miRNA release over 35 days [48]. Furthermore, miRNA release from encapsulated MSNs was GSH-triggered, exhibiting a two-factor release process. Thiolated alginate-PEG diacrylate hydrogels showed good biocompatibility and miRNA release rates [62]. Hydrogels containing PEG with higher molecular weight showed slightly better release rates than those containing lower molecular weight PEG. Using higher molecular weight PEG in the hydrogel also showed decreased hydrophilicity, with a 50% lower swelling rate and a flatter micromorphology. This indicated that PEG hydrogels can be fine-tuned for specific applications, e.g., PEG with higher molecular weight could be used in tissue regeneration where mechanical strength is necessary. Peptide-loaded PEG hydrogels exhibited similar swelling behaviors with good cell viability [51]. Precise timing of miRNA release was also achieved in this study through MMP sensitivity. Physically entrapped miRNA nanoparticles were released primarily through diffusion over 144 h at a steady rate. In contrast, chemically linked miRNA nanoparticles exhibited a small initial release possibly due to physical diffusion. A burst release was observed with the occurrence of MMP7, indicating strong sensitivity.

Polycaprolactone (PCL) is a synthetic, biocompatible polymer that can be employed in thermoresponsive release due to its low melting point [61]. Although these hydrogels are non-toxic with strong mechanical properties, they have slow degradation rates, which restricts their application [63]. PCL electrospun fibers and collagen form a fiber–hydrogel scaffold that achieves stable miRNA release over 90 days [64].

Gelatin is a natural, protein-based biopolymer often modified with methacrylic anhydride to form gelatin methacrylate (GelMA) for biomedical applications. Furthermore, nanomaterials are also added to improve mechanical strength [26,27,42]. GelMA hydrogels loaded with nanoparticles retained their structural integrity and achieved 100% miRNA release in vivo due to complete hydrogel degradation [42]. These hydrogels have also exhibited good cell viability [27]. GelMA-nano clay hydrogels exhibited sustained miRNA release and improved strength compared to GelMA alone [26].

Peptide-based hydrogels are low-cost and tunable, while also biocompatible and biodegradable in vivo [65]. Self-assembling peptide hydrogels have structures that mimic the natural ECM, making them useful in tissue regeneration applications [39]. Hydrogels formed from the Ac-(RADA)_4_-NH_2_ sequence exhibit 70% miRNA release over 40 days. Peptides have also been incorporated into hydrogels to confer additional functions, such as antimicrobial properties [66]. Research on peptide-based hydrogels in miRNA therapeutics is currently in its early stages, with very few reports analyzing miRNA release kinetics. Recently, a novel class of short peptides with tunable mechanical and structural features was reported [67]. N^α^-9-fuorenylmethoxycarbonyl-diphenylalanine (Fmoc-FF) hydrogels are well studied due to their self-assembly kinetics and simplicity. They show good cell viability as well as stable drug release [68]. The advantageous properties seen indicate that these hydrogels could be employed in miRNA release studies.

Fibrin is a fibrous protein that has been used in tissue regeneration applications for its stiffness and biodegradability [69]. Fibrin-hyaluronan hydrogels exhibit good cell viability [70]. Release profiles indicate that around 50% of miRNA is retained within the hydrogel after 14 days. However, hydrogels loaded with miRNA/lipofectamine complexes exhibited slower release, with about 85% retained after 14 days. Silk fibroin hydrogels are known for their mechanical strength, non-toxicity, and tunable degradation rate [71]. Silk fibroin hydrogels carrying miRNA-laden exosomes produced better release rates, with 40% retention after 36 days [38].

### 4.2. Scalability and Commercialization

While it is important to develop hydrogel systems that can ensure miRNA stability and efficient cellular uptake, it is equally important to consider manufacturing processes and scalability of the designed systems. For miRNA delivery, where nuclease degradation is a key issue, precise formulation and release are critical for scalable production. Another aspect is the cost-effectiveness of the materials and processes, to ensure therapies remain affordable to patients. The one-pot system is a synthesis method where hydrogel fabrication and miRNA loading occur in a single step. Carthew and coworkers [23] synthesized a gelatin-PEG hydrogel in a one-pot system via a thiol-ene photoclick reaction. While this simple method reduces production time, the rapid photoclick reaction is light-triggered and requires a UV source. This type of external equipment in hydrogel fabrication increases production costs. Hydrogel scaffolds fabricated through 3D printing are an interesting yet costly endeavor [32]. Synthesis methods that use mild reaction conditions will require less energy consumption and can reduce expenses. A PEG-Ag hydrogel was formed using simple dissolution and mixing without a catalyst. Additionally, miRNA loading occurred through a second mixing step [29]. Simple fabrication methods allow for consistent scaling-up in commercialization. A major component in ensuring low costs is the material selected for use in the hydrogels. Natural polymers are biocompatible and easy to source, which translates to cost-effectiveness. Alginate, chitosan, and hyaluronic acid have exhibited stability and sustained miRNA release [44,62,72]. Conversely, synthetic polymers like PEG and PAMAM offer consistency in synthesis, which will further streamline mass production. PEG is also biocompatible and relatively inexpensive, leading to its frequent consideration in hydrogel synthesis [46,48,51].

## 5. Alternative miRNA Delivery Methods

Several systems have been developed for the stable and efficient delivery of miRNA. These include viral vectors, lipid-based carriers, polymeric nanoparticles, inorganic nanoparticles, and exosome-aided delivery.

### 5.1. Lipid-Based Carriers

Lipid-based nanocarriers are well-studied and widely used in gene therapy. Liposomes are excellent miRNA delivery agents due to their biocompatibility and ease of preparation. The commercially available transfection agent, Lipofectamine, is a cationic lipid-based reagent that functions by encapsulating miRNA within the bilayer and fusing with cell membranes for efficient uptake [73]. It often serves as a baseline for comparison with developing therapeutics [74]. However, liposomes can induce cytotoxicity and immune responses. They also suffer from a short half-life that can reduce their therapeutic efficacy [75]. Comparatively, hydrogels have minimal toxicity, which can be reduced based on the design materials and modifications. They also outperform liposomes in providing longer circulation times with sustained release.

### 5.2. Viral Vectors

Lentiviruses, adenoviruses, adeno-associated viruses, and herpes simplex viruses are amongst the most studied viral vectors in miRNA delivery systems. They exhibit high transfection efficiency by integrating miRNA sequences into the host genome and are also known to be highly stable [76]. However, they have been found to possess serious risks. Lentiviruses carry the risk of insertional mutagenesis and oncogenesis, while adenoviruses have high transduction efficiencies but can cause gastroenteritis and conjunctivitis. Adeno-associated viruses elicit weak immune responses and are relatively expensive [75]. The risks associated with viral vectors limit their clinical application and require a rigorous design process. Consequently, high production costs are incurred, and scalability is difficult. Hydrogels offer safety without genetic integration and risk for negative alterations. They can be designed to provide a sustained release that rivals the high transfection efficiency of viral vectors.

### 5.3. Polymeric Nanoparticles

Polymers such as chitosan and PLGA are structurally flexible and can form nanoparticles. They can encapsulate miRNAs, which facilitates controlled release and protection from nuclease degradation. Additionally, they are easily modified with chemical groups that confer them with new functionalities. Synthetic polymers such as PEI are excellent transfection agents but have shown cytotoxicity [73]. PEG is commonly used to enhance the biocompatibility of cytotoxic polymers in a conjugation process known as ‘PEGylation’. Polymeric nanoparticles formed from synthetic polymers often require complex production methods that can be costly [77]. However, this issue extends to hydrogels since synthetic polymers are commonly employed in production. In contrast, hydrogels can be designed to degrade naturally, reducing long-term risks. They also offer injectability, maximizing the therapeutic effect and reducing potential tissue damage [39].

### 5.4. Exosome-Based Delivery

Exosomes are well-known natural vectors superior to synthesized nanoparticles in that they have a longer circulation time within the body [76]. As a natural vector, they are efficient at internalization due to exosome-specific communication pathways [73]. In a study on miRNA delivery, loaded exosomes induced significant anticancer effects in vivo [78]. A challenge facing this delivery system is scalability. Mass production of exosomes with reliable quality is complex and costly. On the other hand, hydrogels do not require complex isolation methods and can be easier to produce with other natural materials.

## 6. Applications

The most common applications of hydrogel-based miRNA delivery systems are geared toward cancer, tissue regeneration, wound healing, and cardiovascular diseases (Table 1). Henceforth, this section will be focused on the topics mentioned above.

### 6.1. Cancer

In treating hepatocellular carcinoma (HCC), alginate/polyethyleneimine hydrogels carrying miRNA-192 exhibited an enhanced tumor inhibitory effect compared to the miRNA alone. β-catenin and c-MYC were downregulated under this hydrogel system, presumably by suppressing the GSK3β/Wnt/β-catenin signaling pathway [24]. Triple-negative breast cancer (TNBC) has been the focus of much of the research involving hydrogel-based miRNA delivery systems due to the poor prognosis surrounding this aggressive cancer. The current conventional treatments for TNBC are limited by poor cellular uptake, bioavailability, and resistance. Self-assembled RNA nanohydrogels are gaining momentum in TNBC treatment. However, combining gene therapy, chemotherapy, and photodynamic therapy has yielded promising results. Hydrogels loaded with miRNA, the chemotherapy drug doxorubicin, and the photosensitizer chlorine e6 were shown to synergistically inhibit metastasis and improve the hypoxic environment of the tumor [41]. Similar synergistic results were observed when oncogenic miRNA-21 was delivered with doxorubicin via a DNA nano hydrogel. Significant blockage of the miRNA was observed along with significant increases in mRNA expression of PTEN and PDCD4 genes, which are known tumor suppressor genes [79]. The RNA triple-helix hydrogel is also a relatively new strategy showing tumor size reduction without toxicity-related inflammation [19]. This strategy has also been employed in non-small cell lung cancer and solves the issue of chemoresistance through gene silencing for both pump and non-pump resistance to chemotherapy. The RNA triple-helix hydrogel treatment has been reinforced by adding doxorubicin and TMPyP4, creating an advanced combination therapy with targeted inhibition of cancer cell proliferation [43]. Zheng and coworkers [45] developed a nucleolin receptor-targeting aptamer in a hydrogel system with fluorescence tracking, by incorporating near-infrared DNA-templated CdTeSe quantum dots. GSH and miRNA were incorporated into the hydrogel system and were released in a controlled manner. For early diagnosis, Seo and coworkers developed a novel nucleic acid amplification circuit-based hydrogel system for highly sensitive detection of metastatic gastric cancer-derived exosomal miR-21 and miR-99a. In this one-step reaction, DNA amplification occurred within the hydrogel via rolling circle amplification followed by fluorescent labeling for detection [80]. Table 1 summarizes the different hydrogels used in miRNA delivery for cancer.

**Table 1:** A summary of the material used to formulate hydrogels for cancer applications and the miRNA used.

miRNA	Hydrogel Formulation	Application	Refs.
AgomiR-205; antimir-205; antimiR-221	PAMAM G5	Triple-negative breast cancer	[22]
miR-29b	Hyaluronic acid-PEG	Osteosarcoma; tumor theranostics	[35]
miR-96; miR-182	PAMAM G5 (AuNP)	Breast cancer	[54]
miR-205antimiR-221; let-7a miR-34a; miR-145	Self-assembled RNA nano hydrogel	Triple-negative breast cancer; non-small cell lung cancer	[19,43]
miR-205; miR-182	RNA nano hydrogel (MnO_2_ NP)	Triple-negative breast cancer	[41]
miR-192	Alginate-polyethyleneimine	Hepatocellular carcinoma	[24]
miR-21	Chitosan (carbon dots)	Bioimaging	[44]
miRNA	CMC chain (CdTeSe quantum dots) and anti-nucleolin aptamer-modified CMC	Tumor theranostics	[45]
miR-21; miR-99a	PEG- diacrylate	Gastric cancer detection	[80]

### 6.2. Tissue Regeneration

One of the most widely studied fields in hydrogel-mediated miRNA delivery is tissue regeneration. Hydrogels are particularly suited to bone regeneration, as they can uniquely take on the shape of complex defects. They can protect cells during gene delivery and support osteogenesis by creating a favorable microenvironment [23]. Autografts and allografts, currently the most used treatment for bone defects, suffer from limitations such as graft absorption, secondary infection, and fractures [46]. Three-dimensional (3D) printing of hydrogels has become increasingly popular in bone regeneration for precise design and fabrication of a scaffold meeting optimized requirements for the application. These plotting techniques have been used to fabricate gelatin-alginate hydrogel scaffolds for sustained miR-29b release [32]. Drug and gene co-delivery has also been studied in tissue regeneration with simultaneous miRNA and aspirin delivery using thermoresponsive hydrogels introduced into a favorable microenvironment. This promoted neurogenesis and bone regeneration [48].

PEG and gelatin are common polymers used in cartilage engineering, presumably due to their adjustable mechanical strength and UV-cross-linking capabilities. In intervertebral disc degeneration (IVDD), a type of osteoarthritis, biomaterials with softness and elasticity are required due to the continuous pressure cartilage in the spinal column undergoes. In cartilage regeneration, materials with high mechanical strength, such as plastics and metals used in surgical treatment, may damage neighboring tissue and cause undesirable secondary damage [29]. One major limitation to cartilage regeneration is the limited self-regenerative capacity of cartilage itself. Extracellular vesicles, known for their high stability and rich sources, carrying miR-23a-3p encompassed within a gelatin-nano clay hydrogel, were able to promote cartilage regeneration via the PTEN/AKT signaling pathway [26]. Due to its prevalence among adults and severe symptoms, osteoarthritis has been widely studied in hydrogel-based therapies [27,39,40]. It was reported that using stem cell-homing hydrogels to deliver aging-related miR-29b-5p synergistically led to cartilage repair and chondrocyte regeneration [39]. Table 2 summarizes the different hydrogels used in miRNA delivery for tissue regeneration.

### 6.3. Wound Healing

Wound healing is a complex process often associated with adverse effects such as inflammation and scar formation. Hence, it is critical to maintain the integrity of the skin. Healing is a physiological process that includes cell proliferation, hemostasis, inflammation, and remodeling [9]. The current conventional treatments for wounds, such as sutures, cause localized stress and carry the risk of infection. Hydrogel-miRNA therapies are beneficial, as they can physically protect wounds whilst simultaneously releasing miRNA for a wide array of functions, such as increasing the expression of anti-inflammatory genes and decreasing the expression of proinflammatory markers [42], suppressing collagen deposition for scarless wound healing [62], and increasing expression of angiogenic factors [55]. Exosomes have also been advantageously incorporated into miRNA-hydrogel systems due to their anti-inflammatory functions. A novel treatment for damaged corneal epithelium has been proposed using modified hyaluronic acid hydrogel releasing miR-24-3p-rich exosomes. Enhanced epithelial healing, fibrosis inhibition, and macrophage activation indicated a promising therapeutic system [49]. Novel cryogels have also been investigated for enhanced wound healing. A cryogel formulated using chitosan, citric acid, and silver NPs showed significant antibacterial activity and wound healing in vivo [82]. Table 3 summarizes the different hydrogels used in wound healing.

### 6.4. Cardiovascular Diseases

Myocardial infarction, a type of cardiovascular disease, has been a leading cause of death and often leads to chronic heart failure [30]. Many treatments are geared toward the symptoms instead of inducing tissue repair [46]. As such, many researchers aim to provide treatment strategies that enhance angiogenesis of the affected area [6,30,47]. A modified hyaluronic acid hydrogel encapsulating miR-199a-3p was designed to restore the affected myocardium’s contractility and reduce scar size, improving cardiac function [47]. Li and coworkers achieved increased vascularization and decreased infarct size by applying a polyethylene glycol hydrogel encapsulating mesoporous silica NPs carrying miR-21-5p [30]. Vascular disease is an age-induced disease with no established treatment. It was reported that miR-675, which is known to promote skeletal muscle regeneration, prevents vascular disease via the TGF-β1/p21 pathway. The authors proposed that a silk fibroin hydrogel encapsulating miR-675 exosomes is a promising treatment for many diseases [38]. Table 4 summarizes the various hydrogels used in treating cardiovascular diseases.

## 7. Future Perspectives

While miRNA therapeutics and hydrogel systems hold great potential, there is still much to be done in research so that successful treatments can be approved for use. Even though research has been undertaken in various fields to develop potential miRNA treatments, the mechanism of cellular uptake is still not fully understood. This is particularly important in determining how long-term usage of miRNA therapeutics will impact the cell cycle and overall health. Although positive results have been observed with hydrogel systems as carriers for miRNA, several challenges still need to be overcome before these treatments can be translated to the clinics—namely, the potential off-target effects and the ability of a single miRNA inhibitor for cross-family miRNA inhibition. Current in vivo research does not look at potential off-target effects nor have long-term studies been performed to analyze safety. Another issue is acquiring scalable manufacturing processes and ensuring batch-to-batch consistency. The high costs associated with scaling up and the lack of evidence for efficacy reduce interest in pursuing these therapies in clinical trials. Commercialization can also be complicated due to the need for licensing and intellectual property rights.

Research into RNA triple helix hydrogels is a fascinating area that requires future attention since different miRNA sequences can be used to target a wide array of diseases. Combining this with selected targeting ligands and aptamers may create targeted treatments for various health issues. Several studies geared toward combination therapy that involves the synergism between miRNA therapeutics, chemotherapy, and immunotherapy have yielded positive results. This might indicate that establishing a clinically approved miRNA treatment lies in a combined approach instead of a singular miRNA delivery system. Unlike siRNA therapeutics, miRNA has not achieved similar success, and innovative approaches must be considered to make advancements in this field. However, combined therapy research has encountered limitations, and researchers must continue fine-tuning the system. Another aspect of hydrogel-based systems that needs to be elucidated is the optimal time frame for therapeutic administration. In the case of chronic illnesses, long-term safety and efficacy must be ensured.

Another major obstacle to the clinical development of miRNA drugs is the glaring lack of research in large-animal models. Large-animal studies are necessary before continuing human trials. This issue extends from cardiac diseases to cancer, where in vivo work has primarily been undertaken on small animals such as mice. The limitation of the mouse model is that inflammatory reactions have been recorded in myocardial infarction treatments, creating additional damage. It is necessary to determine whether adverse reactions result from injectable hydrogels or are a limitation of the model. Future research must progress to larger models, such as pigs, which are useful, as they are anatomically and physiologically similar to humans. Lastly, further optimization of hydrogel-miRNA formulations and research into their interactions is required since alteration of the gel characteristics may alter the miRNA release profile. These findings can provide information on conditions for increased transfection efficiency and retention and will help direct future work.

## 8. Conclusions

The delivery of miRNA using hydrogel systems has certainly shown significant advancements in attaining sustained in vivo release. Where other vectors such as lipids, peptides, polymers, and NPs have fallen short, hydrogel delivery has seemingly emerged as a worthy alternative. Hydrogel systems have been fine-tuned to elicit triggered release of miRNA, allowing for precise delivery. Furthermore, these systems have shown little to no toxicity for the most part and have achieved the desired results in several applications. Indeed, there is still essential research yet to be undertaken in fully understanding the mechanisms involved in miRNA uptake and completing large-animal studies. However, the outstanding potential of miRNA via hydrogel systems is a promising area of research that deserves attention.

## Figures and Tables

**Table 2:** A summary of the materials used in hydrogels for tissue regeneration and the miRNA sequences used in each system.

miRNA	Hydrogel Formulation	Application	Ref.
miR-100-5p; miR-143-3p	Gelatin-norbornene-PEG	Bone-tissue engineering	[23]
AgomiR-874	PEG-Ag	Cartilage regeneration	[29]
AntimiR-874-5p; antimiR-467a-3p	Sodium alginate-pluronic F127 (extracellular vesicles)	Muscle regeneration	[31]
miR-26a	PEG	Bone regeneration	[46]
miR-214	Decellularized ECM	Tissue regeneration	[37]
AntimiR-23a-3p	Gelatin-nano clay (extracellular vesicles)	Cartilage regeneration	[26]
miR-222	PEG- PLGA- PNIPAM (MSN)	Bone regeneration	[48]
miR-140	Gelatin-methylacroyl-modified polyamidoamine	Cartilage regeneration	[27]
antimiR-221	Fibrin-hyaluronan	Tissue regeneration	[70]
miR-29b	Gelatin-alginate (AuNP)	Bone regeneration	[32]
miR-29b	Chitosan (graphene oxide NP)	Bone regeneration	[53]
miR-126; miR-146a	Alginate (exosomes)	Cardiac regeneration	[33]
AntimiR-21	Gelatin-phenylboric acid-cyclodextrin	Tissue regeneration	[81]
AntimiR-21	Glycidyl methacrylate-carboxymethyl chitosan (tannic acid NPs)	Intervertebral disc regeneration	[50]
miR-155	PEG-MMP7-sensitive peptides (NPs)	Bone regeneration	[51]
miR-99b-3p	Hyperbranched PEG diacrylate-hyaluronic acid (exosomes)	Cartilage regeneration	[40]
miR-132; miR-222; miR-431	Polycaprolactone-collagen	Nerve regeneration	[64]
AgomiR-29b-5p	Self-assembling peptide-bone marrow-homing peptide motif	Cartilage regeneration	[39]

**Table 3:** A summary of the materials used in hydrogels for wound healing applications and the miRNA sequences employed.

miRNA	Hydrogel Formulation	Application	Ref.
miRNA	Hyaluronic acid-polyarginine	Antibacterial and anti-inflammatory	[75]
miR-223-5p	Gelatin-methacryloyl (HA NP)	Increased anti-inflammatory gene expression and decreased pro-inflammatory markers	[42]
miR-29b-3p	Thiolated alginate-PEG diacrylate	Angiogenesis promotion and collagen deposition	[62]
miR-24-3p	Hyaluronic acid-di(ethylene glycol) mono methyl ether methacrylate	Corneal epithelial healing	[49]
AntimiR-26a	Collagen (polyethyleneimine ceria nanocluster)	Accelerated diabetic wound closure	[55]
AntimiR-708-5p	Hyaluronic acid-adipic dihydrazide and hyaluronic acid-quaternary ammonium-aldehyde	Antibacterial and pro-osteogenic differentiation	[59]
miR-21-5p	Hyaluronic acid-polydopamine-DP-7 (exosomes)	Antibacterial wound closure	[66]

**Table 4:** A summary of the materials used in hydrogels for treating cardiovascular diseases and the miRNA sequences utilized.

miRNA	Hydrogel Formulation	Application	Refs.
miR-19b; antimir-15 antimir-1; antimir-195	UPy-PEG	Controlled release study; Cardiomyocyte proliferation	[15,52]
miR-675	Silk fibroin (exosomes)	Aging-induced vascular dysfunction	[38]
miR-21-5p	PEG (MSN)	Anti-inflammatory and proangiogenic effects for myocardial infarction	[30]
AntimiR-92a	Deoxycholic acid-modified polyethyleneimine polymeric conjugates	Ischaemic heart disease	[6]
miR-146a	Chitosan (PEG-PLA NP)	Allergic rhinitis	[25]
miR-214	HPMC-C_12_ (PEG-b-PLA NP)	Cardiovascular diseases and cancer	[28]
miR-302b; miR-302c; miR-29b	Cyclodextrin β-modified hyaluronic acid-adamantane-modified hyaluronic acid	Myocardial infarction; Renal interstitial fibrosis	[7,36]
miR-199a-3p	Elastin-like protein-hyaluronic acid (PFBT NPs)	Myocardial infarction	[47]

## Data Availability

No new data were created or analyzed in this study.

## Abbreviations

The following abbreviations are used in this manuscript:

3D	three-dimensional
Agomirs	modified miRNA mimics
CdTeSe	cadmium tellurium selenide
DEGMA	di(ethylene glycol) methyl ether methacrylate
ECM	extracellular matrix
Fmoc-FF	N^α^-9-fuorenylmethoxycarbonyl-diphenylalanine
GelMA	gelatin methacrylate
GMC	glycidyl methacrylate
GSH	glutathione
HA	hyaluronic acid
HCC	hepatocellular carcinoma
HCV	hepatitis C virus
HPMC-C_12_	hydroxypropylmethylcellulose
IVDD	intervertebral disc degeneration
LAP	lithium phenyl-2,4,6-trimethylbenzoylphosphinate
MAL	maleimide
MDA-MB-231	human breast cancer cell line
miR-chol	cholesterol modified miRNA
mRNA	microRNA
MMP	matrix metalloproteinases
MSN	mesoporous silica nanoparticle
NHS	N-hydroxysuccinimide
NPs	nanoparticles
NSCLC	non-small-cell lung cancer
OEG	oligo-ethylene glycol
p21	cell-cycle regulator protein
PAMAM	polyamidoamine
PCL	polycaprolactone
PDCD4	programmed cell death protein 4
PEG	polyethylene glycol
PEG-*b*-PLA	poly(ethylene glycol)-block-poly(lactic acid)
PFBT	polyfluorene-alt-benzothiadiazole
PEI	polyethyleneimine
PLGA	poly(lactic-co-glycolic acid)
PNIPAM	poly(N-isopropyl acrylamide)
PNP	polymer-nanoparticle
PPG	polypropylene glycol
PTEN	phosphatase and tensin homolog
PTEN/AKT	signaling pathway involved in cellular growth
RCT	rolling circle transcription
RISC	RNA-induced silencing complex
RNA	ribonucleic acid
RNAi	RNA interference
ROS	reactive oxygen species
SAP	self-assembling peptide
SDS	sodium dodecyl sulfate
SH	sulfhydryl group
TGF-β1	transforming growth factor beta 1
TMPyP4	5,10,15,20-Tetrakis-(N-methyl-4-pyridyl) porphine
TMTME	too many targets for miRNA effect
TNBC	triple-negative breast cancer
UPy	ureido-pyrimidine moieties
UTR	untranslated region
UV	ultraviolet
